# Comment Concerning the Effects of Light Intensity on Melatonin Suppression in the Review “Light Modulation of Human Clocks, Wake, and Sleep” by A. Prayag et al.

**DOI:** 10.3390/clockssleep3010011

**Published:** 2021-02-10

**Authors:** Peter Bracke, Eowyn Van de Putte, Wouter R. Ryckaert

**Affiliations:** ESAT-WaveCore, Light & Lighting Laboratory, KU Leuven, 9000 Ghent, Belgium; peter.bracke90@telenet.be (P.B.); eowyn.vandeputte@ugent.be (E.V.d.P.)

## Abstract

Dose-response curves for circadian phase shift and melatonin suppression in relation to white or monochromatic nighttime illumination can be scaled to melanopic weighed illumination for normally constricted pupils, which makes them easier to interpret and compare. This is helpful for a practical applications.

The excellent review “Light Modulation of Human Clocks, Wake, and Sleep” by A. Prayag et al. in *Clocks & Sleep* (2019) [1] gives an overview of the effects of i.a. timing, duration, intensity, and spectrum of light on non-image forming functions, which are mediated by the intrinsically photosensitive retinal ganglion cells (ipRGCs) in the retina.

A review is not only of interest to the science community, but also provides valuable information for those wishing to apply lighting for wellbeing: the lighting industry and the interested end users. However, Figure 5 ‘Melatonin suppression threshold and saturation as a function of melanopic lux, in humans’ could be misinterpreted by this audience, as if monochromatic light sources need less melanopic weighed irradiation than white light sources for the same response. This is not the case, since the data for the monochromatic light sources is for pharmacologically dilated pupils and with normal pupil constriction (as for the white light response curves in Figure 4), a much higher melanopic irradiance will be required for the same monochromatic response. A misinterpretation could, in the worst case, lead to someone from the lighting industry or an end user applying low level monochromatic light as a circadian boost for people spending most of their daytime indoors, referring to the review as the scientific reference. By converting the illuminance in Figures 4 and 5 into melanopic equivalent daylight illuminance for non-dilated pupils, the risk of misinterpretation could be minimized.

There are dose–response relationships for circadian phase shift [2], melatonin suppression and alertness [3] in relation to nighttime illumination (Figure 4). There is a lot of interest for lighting that supports our evolutionary fit: sleep at night and active during the daytime (diurnal). However, for daytime responses (e.g., phase advance, prior light effect, reaction time, etc.), the results are most often limited to two values of irradiation [4].

The spectral dose–response relationship has been quantified for melatonin suppression for eight wavelengths after 1.5 h light stimulation in a ganzfeld with pharmacologically dilated pupils [5]. A dose–response relationship for a 420 nm wavelength was reported separately [6]. Figure 5 of the review shows that this fits very well with a melanopsin weighed irradiance [7]. However, at these melanopsin weighed irradiances, pupils normally are constricted. For normally constricted pupils, the corneal irradiance needed for the same ipRGC irradiance will be higher, corresponding to the inverse ratio of pupil areas (average dilated pupil diameter in the experiments was 7.19 mm). Based on the pupil size in function of the melanopic equivalent daylight illuminance (see annex), the photon density and ‘melanopic illuminance’ can be converted to melanopic equivalent daylight illuminance for non-(pharmacologically) dilated pupils.

The conversion is preferably done by starting from the melanopic equivalent daylight illuminance for non-dilated pupils (as an example: 154 lux) for which the average pupil diameter at 24.5 y can be calculated (for this example: 2.524 mm). The same ipRGC irradiance for a dilated pupil of 7.19 mm is achieved with a melanopic equivalent daylight illuminance × d^2^/7.19^2^ (e.g., 154 × 2.524^2^/7.19^2^ = 18.98 lux). The corresponding ”melanopic illuminance” is the melanopic equivalent daylight illuminance × 1.104 (e.g., 18.98 lux × 1.104 = 20.95 lux). The corresponding 9 wavelength melatonin suppression for this dilated melanopic illuminance is calculated by the logistic function from Prayag et al.: 1/(1 + (x/20.95)^0.82^). This conversion is done for a range of melanopic equivalent daylight illuminance between 1 and 10,000 lux (Figure A1).

For the same melatonin suppression, there is 2.2× more melanopic weighed illuminance required for a single wavelength than for white light. In the white light condition the melatonin suppression was assessed after 3 h vs. 1.5 h for the single wavelength, but many other studies have shown that the melatonin suppression for white light is the same after 1.5 h and 3 h of nighttime illumination. It could be that the intrinsic melanopsin-mediated response of the ipRGC might be more complex than a linear spectral summation. Still, a linear spectral summation approach does predict the effect of tuned spectrum quite well [8].

Spectra can be made with a different melanopic equivalent daylight illuminance for the same (photopic) illuminance and the same white color (CCT: correlated colour temperature, x,y-values). A study [9] was done on the effect of the melanopic/photopic ratio (MDER: melanopic daylight efficacy ratio) of white light in the late evening (same photopic illuminance, same CCT, slightly different x,y). This study also measured the melatonin suppression, but it differed from the previous studies in that the light condition was applied before the melatonin onset (evening) and not after the melatonin onset (night). The last melatonin measurement was also at an earlier time than those reported in the previously mentioned studies, and there is no baseline reference, so the results cannot be correctly compared. Still, the melatonin suppression at 171 lux melanopic equivalent daylight illuminance (≈57%) is very close to the value on the above curve for white light, whilst the melatonin suppression at 49 lux melanopic equivalent daylight illuminance (≈22%) is less than on the above curve.

Another study [10] did not find a difference between tuned spectra, but the values of the melanopic equivalent daylight illuminance of 388 lux and 220 lux should indeed only give a very small difference according to the above curve.

This confirms the takeaway message of Figure 5 of the review: the melanopic weighing is most appropriate and adequate for ipRGC mediated responses. However, readers could be misled when comparing Figures 4 and 5 of the review. It is worthwhile to clearly state in the review that the illuminance of Figure 4 and Figure 5 cannot be compared, mainly since Figure 5 is not with normal pupil constriction. Readers could be helped in making the comparison by indicating that the illuminance in Figure 5 should be multiplied by 5× to 10× for normal pupil constriction. Or Figures 4 and 5 of the review could be expressed in ‘melanopic equivalent daylight illuminance for non-dilated pupils’ as shown in Figure 1 of this comment.

## ANNEX

Converting the data to melanopic irradiances for normally/constricted pupils.

The CIE (International Commission on Illumination) has introduced a useful standard S-026 for the metrology of ipRGC influenced responses to light [11]. A spectrophotometer that measures the melanopic equivalent daylight illuminance (MEDI) is currently the most practical instrument to be used in the field (cost, availability). Prior lab and field results are not expressed in this quantity. There are dose–response curves for the melatonin suppression, phase shift, pupil size, etc., in relation to the corneal irradiation (or equivalent illuminance). These are typically sigmoid curves that can be fitted with a logistic function. 

## Dose–Response Curves for Circadian Phase Shift and Melatonin Suppression vs. MEDI

The fit of a logistic function is tabulated in the paper by Zeitzer et al. [2]. The dose–response curves can be easily converted to a normalized response in melanopic equivalent daylight illuminance (relative to the dark response and maximal light response). The illumination used in the experiments were Philips 6500 K fluorescent lights, which have a melanopic daylight efficacy ratio MDER of 0.85. The illumination values have to be multiplied by 0.85 to have the dose–response curves in melanopic equivalent daylight illuminance.

The parameters from the four parameter logistic function f(x) = (a − d)/(1 + x/b)^c) + d can be used directly to create the relative response in melanopic equivalent daylight illuminance: f_rel_(x) = 1/(1 + (x × MDER/b)^c).

## Pupil Size vs. MEDI

The pupil area for the time period of interest (5 min–2 h) has a spectral sensitivity that corresponds well with the melanopic [12,13,14], weighing and depends on the product of radiance × area (deg^2^) [15,16].

The simplified formula [15] for the pupil diameter:

$$D_{U}=\frac{18.5172+0.122165\times f-0.105569\times y+0.0001386645\times f\times y}{2+0.0630635\times f}$$

with *y* = age in years and *f* = (L_v_ × a)^0.41^.

**Figure A2:**
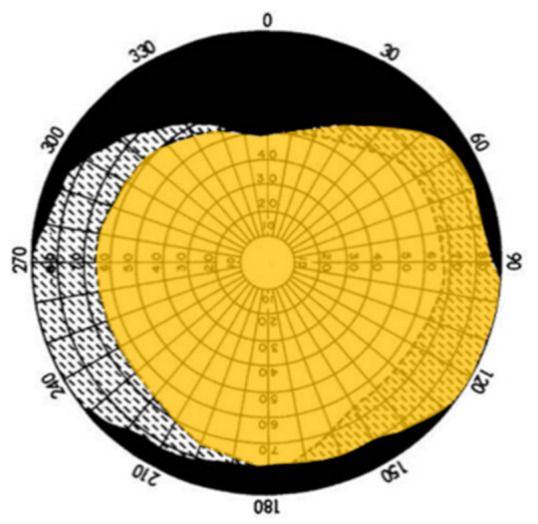
The human visual field [18] for indoor photopic binocular vision. Yellow area for the right eye is 16,200 deg^2^.

The normal achromatic visual field is 94° temporal, 62° nasal, 50–55° upwards and 70–80° downwards [17]. The field area of one eye is approximately a = 16,200 deg^2^ (based on Figure A2 [18]). The field area that is only visible to one eye should be scaled by 0.1 to calculate *f*. For a ganzfeld an equivalent area of 13,500 deg^2^ can be used.

**Figure A3:**
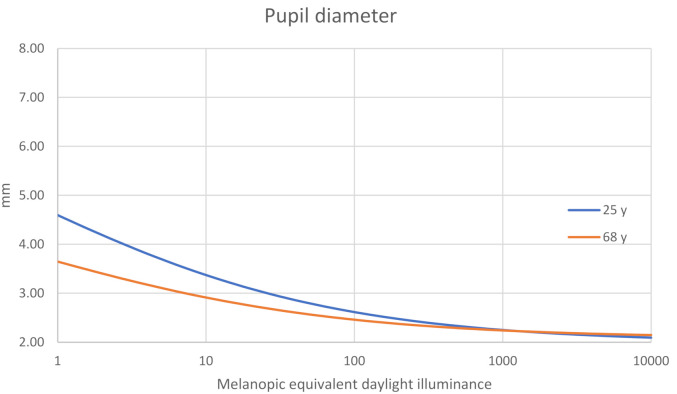
Pupil diameter for ages of 24.5 and 68 year according to the Watson and Yellott simplified unified formula.

L_v_ in the above formula is the photopic luminance (cd/m^2^). The formula was fitted to the Stanley and Davies [19] data, but it is not clear what spectrum the light source had in their experiments (e.g., incandescent bulb, xenon projector, etc.). I used a value for the melanopic daylight efficacy ratio (MDER) of 0.5 (Figure A3). For the daytime illumination range, the impact of this assumption on the pupil area is very small.

For a ganzfeld: Luminance = illuminance/π Radiance = irradiance/π L = E/π

L_v_ = E_v_/π = E_mel,v_^D65^/γ_mel,v_^D65^/π (MEDI/MDER/π)

Thus *f* = (8594·E_mel,v_^D65^)^0.41^

E (W/m^2^) = π·L (W/m^2^/sr)·τ ·D_u_^2^ (mm^2^)/4f_1_^2^ (mm^2^)

f_1_ = focal length = 16.67 mm

“Use of this equation near the visual axis is straightforward. In the peripheral field, however, complications arise. With increasing field angle, the entrance pupil appears as an ellipse of increasing eccentricity and reduced area. Moreover, due to the retina lying on the curved surface of the eyeball, both the distance between the exit pupil of the eye and the retina and the retinal area corresponding to the image of an object area of constant angular subtense diminish with field angle” [20]. These pupil and retinal effects tend to largely compensate one another. Based on data from the wide angle model [21], the total retinal irradiance for the ganzfeld is 95.3% of what the above formula indicates. 

## Single Wavelength Dilated Pupil Irradiance Converted to MEDI for Non-Dilated Pupil

The fit of a logistic function for the 460 nm data is tabulated in the paper by Brainard et al. [5] with the irradiance expressed as a number of photons.

For an x-axis in melanopic equivalent daylight illuminance E_mel,v_^D65^ (MEDI) for non-dilated pupil:

E_non-dilated_ (W/m^2^) = E_mel,v_^D65^(lux)/s_mel_(λ)·*K*_mel,v_^D65^(W/lm)

*K*_mel,v_^D65^ = 1.3262 × 10^−3^ W/lm and s_mel_(λ) = 0.70805 is tabulated in CIE S 026 and should be corrected for the average age in the experiment of 24.5 y (×1.078)

For the same retinal illuminance for a dilated pupil:

E_dilated_ (W/m^2^) = E_mel,v_^D65^(lux)/s_mel_(λ)·*K*_mel,v_^D65^(W/lm)·Du^2^ (mm^2^)/7.19^2^ (mm^2^)

Du is a function of the MEDI as calculated before.

The photon density:

N_p_ = E·λ/(h·c) = E_dilated_ [W/m^2^]·λ [m]/(1.98645 × 10^−25^) [J/s·m/s]

The relative melatonin suppression: = 1 − 1/[1 + (N_p_/8.29 × 10^12^)^1.23^].

## About the Cone Contribution to the Spectral Response

The M1 ipRGCs show cone-driven input [22]: an (L + M) cone-mediated ON response opponent to an S cone-mediated OFF response in primates. As is usual for retinal ganglion cells, the (cone-mediated) receptive field diameters are such that the receptive fields fill the retina without ‘blind’ spots and with minimal overlap per nucleus. The fovea has no ipRGCs. Because of the low density of ipRGCs in the retina, these are receptive fields with a 400–900 µm diameter corresponding to visual angles of 1.4–3.2°.

Rodents are different in having an S cone-mediated ON response with an (L + M) cone-mediated ON response. Conclusions concerning spectral responses from rodent studies—specifically concerning blue light—do not translate directly to primates.

The (L + M) cone mediated response to a maintained light step is transient and declines rapidly. In a ganzfeld experiment, the non-transient responses (>2 min) will be as good as only melanopsin driven. 

## Spectral Response of Circadian Phase Shift

**Figure A4:**
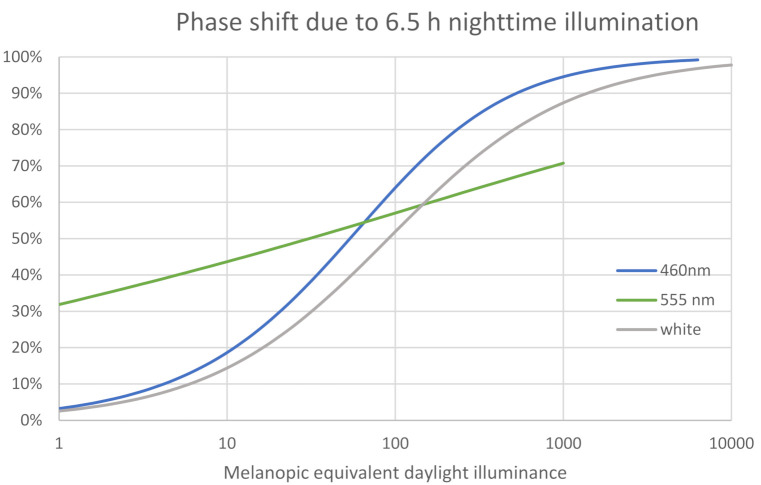
Normalized circadian phase-shift as a function of melanopic equivalent daylight illuminance with non-dilated pupils for a 6500 K fluorescent 6.5h nighttime illumination and for 460 nm and 555 nm illumination.

There are no spectral dose–response studies for phase shift by daytime illumination (synchronization). However, there are spectral studies for nighttime illumination [23]. Unfortunately, the paper did not indicate the average pupil diameter for the dilated pupils, so the above value of 7.19 mm was used for the conversion (Figure A4).

The circadian phase shift due to nighttime light exposure with a 460 nm illumination corresponds well to the 6500 K fluorescent illumination. There is a maximum of 19 minutes difference in phase shift. However, the circadian phase shift due to nighttime light exposure with a 555 nm illumination is very different. There is no model proposed for this. There might also be an exposure time effect. The melatonin suppression dose–response curve for the 555 nm illumination had a much steeper slope after 4 h. The phase shift for the 555 nm illumination might be due to other signals/cues for the circadian system than just the ipRGC signal. We have not evolved, as a species, to have correct responses to monochromatic light and the effects of monochromatic light sources or light sources, which have a high visual response with a very low melanopic response, should be studied extensively before being applied in the field.

## Spectral Response of Pupil Constriction

The rods become saturated at an irradiance level where the melanopsin starts generating an intrinsic response. The spectral response of the rods and the spectral melanopsin driven intrinsic response are quite similar (certainly for daylight CCTs). The rod + melanopsin response is continuous over a very wide dynamic range. Still, with a large irradiance step, there is a transient response of the pupil constriction that is partially driven by the cones [12,13].

There is an L+M-S contribution to the pupillary light response, which is quite small for normal illumination levels and which decreases over the illumination duration [12,14,24] and becomes negligible about 5 min after light onset.

## Inter-Individual Pupil Size Differences

The irradiance on the retina and, thus, the signal from the ipRGC depends on the pupil area. The inter-individual differences in the pupil area should not be neglected. The standard deviation of the pupil area is approximately the same for all light levels and ages: 25–30% [25,26,27,28]. This means that the midpoints of dose–response curves will be individually different: inversely proportional to the pupil area. The slope parameter of the sigmoidal fit stays the same. It has indeed been shown that the inter-individual difference in melatonin suppression by light correlates with the differences in pupil area.

The pupil area decreases with age (but this does not affect the relative pupillary response to light).

## Some Observations

- Spectral summation is not sufficiently statistical proven, but seems sufficiently appropriate.
- Typical viewing conditions differ from a ganzfeld and, thus, there could be a very small L+M-S contribution, but this is negligible unless the lighting itself fluctuates rapidly (peak at 0.2 Hz).
- The formula for pupil size with age in the illumination range of interest is an extrapolation since experimental data is lacking.
- Inter-individual pupil area differences are important (the 90 percentile needs 56% more illumination than the average).
- Many papers are lacking necessary data (such as average pupil area, illumination spectra, etc.).
- A logistic function might not be the physiological correct fit.
- Individual responses in typical living environments with other light spectra could deviate substantially from what the curves predict.

## Figures and Tables

**Figure A1:**
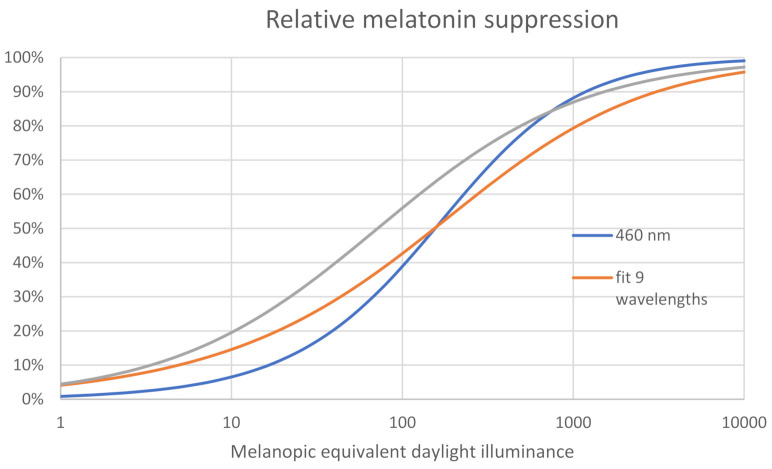
Normalized nighttime melatonin suppression for non-dilated pupils as a function of melanopic equivalent daylight illuminance. The half-maximum is at 71.6 lux for a 6500 K fluorescent illumination, 155.3 lux for 460 nm illumination, and 154 lux for the 9 wavelength fit. The normalization is relative to the dark response and maximal light response to eliminate the difference in measurement methods, test conditions and intra-group differences.

## Data Availability

Not applicable.
